# Reverse Iontophoresis: Noninvasive Assessment of Topical Drug Bioavailability

**DOI:** 10.1021/acs.molpharmaceut.3c00791

**Published:** 2023-12-07

**Authors:** Kieran Moore, Sébastien Grégoire, Joan Eilstein, M. Begoña Delgado-Charro, Richard H. Guy

**Affiliations:** † Department of Life Sciences, University of Bath, Claverton Down, Bath BA2 7AY, U.K.; ‡ L’Oréal Research and Innovation, 1 Av. Eugène Schueller, 93600 Aulnay-sous-Bois, France

**Keywords:** topical bioavailability, reverse iontophoresis, skin, dermatopharmacokinetics, noninvasive drug monitoring

## Abstract

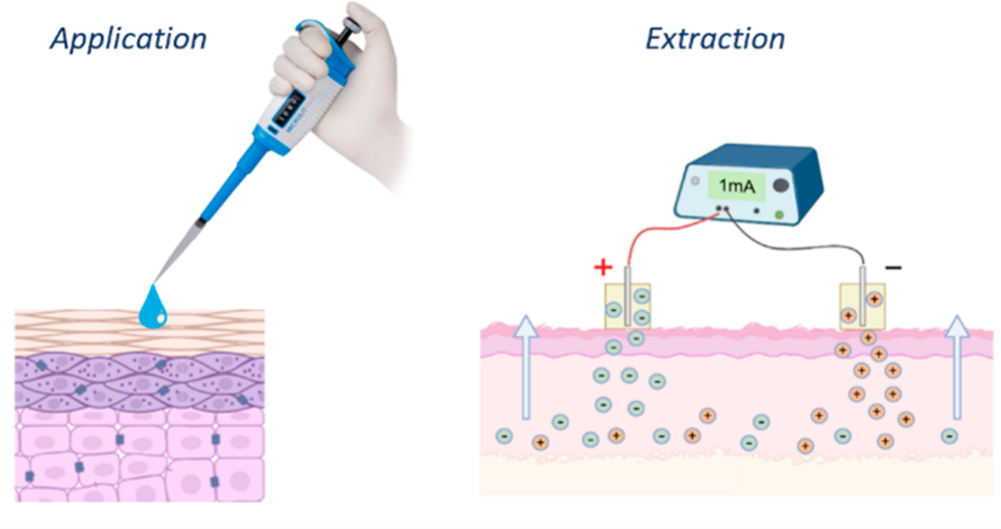

Assessing drug disposition in the skin after the application of a topical formulation is difficult. It is hypothesized that reverse iontophoresis (RI), which can extract charged/polar molecules for monitoring purposes, may provide a noninvasive approach for the assessment of local drug bioavailability. The passive and RI extraction of salicylic acid (SA) and nicotine (NIC) from porcine skin *in vitro* was assessed after a simple solution of the former and a transdermal patch of the latter had been applied for 24 and 8 h, respectively. Immediately after this “passive skin loading”, the amount of drug in the stratum corneum (SC) and “viable” tissue (VT) was measured either (a) after tape-stripping and subsequent solvent extraction of both skin layers or (b) following RI extraction over 4 h. Parallel experiments were then performed *in vivo* in healthy volunteers; in this case, the VT was not sampled and the skin loading period for NIC was only 4 h. RI extraction of both drugs was significantly higher (*in vitro* and *in vivo*) than that achieved passively, and the cumulative RI extraction profiles as a function of time were mathematically analyzed using a straightforward compartmental model. Best-fit estimates of drug amounts in the SC and VT (*A*_SC,0_ and *A*_VT,0_, respectively) at the end of “loading” and two first-order rate constants describing transfer between the model compartments were then determined. The *in vitro* predictions of *A*_SC,0_ and *A*_VT,0_ were in excellent agreement with the experimental results, as was the value of the former *in vivo*. The rate constants derived from the *in vitro* and *in vivo* results were also similar. In summary, the results provide proof-of-concept that the RI method has the potential to noninvasively assess relevant metrics of drug bioavailability in the skin.

## Introduction

The rigorous *in vivo* evaluation of the local bioavailability (BA) of drugs administered topically to the skin is an exacting objective. Of the various techniques that have been considered, the analysis of the drug in skin biopsies^1−3^ or suction blisters^4^ after the application of a topical formulation is considered too invasive, precluding routine use; the skin blanching or vasoconstriction assay is accepted by most regulatory authorities^5−7^ but its restriction to corticosteroids renders a broader application impossible; and while dermal sampling techniques such as microdialysis^8−10^ and open-flow microperfusion^11,12^ are able to generate, in favorable circumstances, concentration–time profiles near the typical target sites of dermatological drugs (*i.e.*, epidermis/upper dermis), these methods are also limited by their invasive nature as well as their technical complexity (a high degree of operator dexterity being required to obtain results that are reproducible, sensitive, and discriminating).

Facile access to the stratum corneum (SC) enables minimally invasive sample acquisition using tape-stripping and is therefore a compelling approach to assess the BA of a drug (**e.g.*,* an antifungal), the site of action of which is within the skin’s barrier layer; indeed, the comparative assessment of objectively determined dermatopharmacokinetic (DPK) parameters related to the rate and extent of SC uptake has been proposed as a surrogate method with which to evaluate bioequivalence (BE) between drug products.^13−15^ More recently, the SC sampling methodology has also been proposed as a tool that can indirectly report on topical drug BA within the skin compartment below the SC;^16−19^ however, although the idea has gained some traction,^20^ its long-term future remains uncertain from a broader regulatory standpoint.^21^ As a result, attention has shifted toward investigation of (in particular) Raman spectroscopic techniques to *noninvasively* and semiquantitatively determine drug concentrations as a function of the depth into skin.^22−24^ Technical issues before any validated approach can emerge are significant; for example, identifying specific Raman signals from the drug of interest that can be clearly distinguished from the background spectrum of the skin, appropriately correcting for signal attenuation at different depths into the skin,^23,25^ and calibrating signal intensity^25,26^ remain incompletely surmounted obstacles. Furthermore, the relatively low sensitivity of Raman spectroscopy precludes its use in the bioavailability assessment of compounds (*e.g.*, potent drugs and cosmetic actives) that are applied sparingly at low concentrations or penetrate the skin slowly.

The work described here investigates a novel implementation of so-called “reverse iontophoresis” (RI) to address the challenge of assessing the local BA of a topically applied drug in the SC and viable epidermal compartments of the skin. RI has been used previously to sample charged and neutral, polar analytes from the interstitial fluid (ISF) in the skin for noninvasive diagnostic^27−30^ and therapeutic drug monitoring^31−33^ applications, and *in vivo* it has been shown that extraction correlates well with the corresponding simultaneously measured blood concentrations. Notably, however, and of direct relevance to the objective of the RI application envisaged here, the initial rates of extraction observed (*e.g.*, for glucose^34,35^ and lithium^31^) are elevated before decreasing gradually to values that correlate with systemic levels. The explanation deduced for this finding is that the skin acts as a “reservoir” within which such substances can accumulate over time. As a result, when RI commences, the analyte of interest must first be “emptied” from the skin before information representative of blood levels can be accessed. A clear demonstration of this phenomenon has been reported for the RI extraction of amino acids from human volunteers.^36^

Clearly, this effect offers an opportunity in the context of assessing the local BA of topically applied drugs. Upon application of RI to skin to which a topical formulation has been previously administered, the drug will first be sampled from the most superficial skin layers and then progressively extracted from deeper tissues. The potential value of the method, in terms of evaluating topical BA, requires deconvolution of the RI extraction data to differentiate between drug localized to the SC and that which has reached the deeper layers. The initial aims of the proof-of-concept research in this paper were therefore (i) to characterize the *in vitro* skin disposition of a drug (*i.e.*, its levels in the SC, underlying skin, and subdermal fluid) following a period of topical exposure and (ii) to then demonstrate the iontophoretic extraction of topically delivered active from the skin. The next objective was to develop an empirical compartmental model and to determine the contributions of the RI extraction profiles from SC- and viable tissue-derived drug. The final goal was to assess the applicability of the RI protocol and modeling approach *in vivo* in human volunteers.

Two drugs, salicylic acid (SA) and nicotine (NIC), that have distinct physicochemical properties and are delivered to the skin in different types of formulations were selected for this study. SA is of cosmetic interest^37^ and of clinical relevance (its licensed indications including the treatment of lesions caused by human papillomavirus (HPV),^38^ muscular pain,^39^ and dry scalp conditions^40^). It has a relatively low molecular weight (138 Da) and a moderate lipophilicity (log [octanol/water] = 2.3^41^), physicochemical properties consistent with reasonably good skin permeation behavior.^42^ SA is a weak acid, negatively charged (p*K*_a_ ∼2.9^41^) at physiological skin pH, and a good candidate, therefore, for reverse iontophoretic extraction.^43^ NIC, of course, is formulated as a transdermal patch for smoking cessation;^44^ additionally, although of a similar size to SA (molecular weight of NIC is 162 Da), NIC is less lipophilic (log [octanol/water] = 1.17^45^), has a relatively high solubility in water at neutral pH, and is considered to be an “excellent” skin penetrant.^46^ NIC is a weak base and is predominantly positively charged (p*K*_a_ ∼8.0) at pH 7.4.^47^

## Materials and Methods

### Chemicals

Salicylic acid (SA, ≥99.5%), sodium salicylate, (−)-nicotine (NIC, ≥99%), ethanol, propylene glycol, sodium lauryl sulfate (SLS), sodium gluconate, and reagents for phosphate buffered saline (PBS; NaCl, KCl, Na_2_HPO_4_, and KH_2_PO_4_) were obtained from Merck (Poole, UK). All electrode materials were obtained from Merck (Poole, UK). Deionized water (resistivity ≥18 MΩ·cm) was generated in a Milli-Q system (Bedford, MA).

### Electrodes

Silver–silver chloride (Ag/AgCl) cathodes were prepared by dipping a Ag wire (0.5 mm diameter) in molten AgCl and were then ready for immediate use. To make the anodes, the AgCl-dipped Ag wire acting as an anode and a platinum wire (0.25 mm diameter), acting as cathode, were immersed in a 50 mM solution of NaCl, and a 0.2 mA current was passed between them overnight.

### Topical SA Formulation and Transdermal NIC Delivery System

SA was formulated extemporaneously at 0.5% (w/v) in a vehicle consisting of water, ethanol (both 47.5% (v/v)), and propylene glycol (5% (v/v)). For *in vitro* experiments, the pH of the formulation was adjusted to 3.0. *In vivo*, to reduce the risk of skin irritation, the vehicle was buffered to pH 4.5. Nicotinell 7 mg/24 h patches (GSK, Brentford, UK) were cut to the area of exposed skin for experiments *in vitro* and to an area slightly greater than that exposed to current for those *in vivo*.

### *In Vitro* Experiments

The methods employed in this series of *in vitro* experiments are described here in detail; for ease of reference, a summary is provided in Table 1.

**Table 1:** Summary of In Vitro Experiments

experiment	description	protocol	rationale
E1	IVPT	1. application of drug formulation^a^	elucidate drug disposition in SC, VT and receptor after topical application
		2. sampling of lower chamber	
		3. surface cleaning	
		4. skin separated into SC and viable tissue (VT)	
E2	IVPT + RI	1. application of drug formulation^a^	determine efficacy of drug extraction by RI after topical application
		2. sampling of lower chamber	
		3. surface cleaning	
		4. period of RI with upper chamber sampling	
		5. skin separated into SC and VT	
E3	IVPT + passive extraction	1. application of drug formulation^a^	determine efficacy of drug extraction by passive diffusion after topical application
		2. sampling of lower chamber	
		3. surface cleaning	
		4. period of passive extraction with upper chamber sampling	
		5. skin separated into SC and VT	
E4	RI from subdermal compartment (SDC)	1. drug introduced into SDC	control experiment to facilitate interpretation of data from E2
		2. period of RI with upper chamber sampling	
		3. skin separated into SC and VT	
E5	Passive extraction from SDC	1. drug introduced into SDC	control to verify that passive extraction from SDC is negligible
		2. period of passive extraction with upper chamber sampling	
		3. skin separated into SC and VT	

a SA solution (0.5% w/v in ethanol/water/propylene glycol) for 24 h. NIC Nicotinell 7 mg/24 h patch for 8 h.

#### Topical Drug Application Procedures

Skin from a female (Large White) pig was acquired within 24 h of slaughter as a waste product of the food industry and was immediately dermatomed (Zimmer, Warsaw, IN) to a nominal thickness of 750 μm, cut into sections, and frozen at −20 °C. On the day of experimentation, the required samples were thawed, and the skin was clamped in vertical Franz-type static diffusion cells (exposed transport area = 2.01 cm^2^) between upper and subdermal chambers (Permegear, Hellertown, PA). The latter was filled with 7.4 mL of 10 mM PBS (pH 7.4; 10 mM Na_2_HPO_4_, 1.8 mM KH_2_PO_4_, 137 mM NaCl, and 2.7 mM KCl) and stirred with a magnetic bar.

For SA, the *in vitro* permeation test (IVPT) procedure involved applying 5.2 μL cm^–2^ of the formulation (corresponding to 26 μg cm^–2^ of SA) to the surface of the skin for 24 h. The Franz cells were thermostatted at 32 °C for the duration of the loading phase (except for brief periods to sample and replace 1 mL of subdermal buffer solution). The IVPT was used to initiate two types of experiments. The first (Table 1, experiment E1) assessed the penetration of SA into the subdermal fluid; at the end of the experiment, the skin was tape-stripped to separate active located in the stratum corneum (SC) and “viable” tissue (VT) below. In the second type, termination of the IVPT was followed by a 4–6 h period of SA extraction by reverse iontophoresis (RI) (Table 1, experiment E2) or passive extraction (Table 1, experiment E3). This was then followed by tape-stripping as performed in experiment E1.

For NIC, a cut section (2.01 cm^2^) of a Nicotinell patch was applied to the skin for 8 h (Table 1, experiment E1), and the IVPT protocol then mirrored the approach used for SA.

#### Tape-Stripping Following Topical Application

At the end of SA application, the skin surface was thoroughly cleaned without disassembling the Franz cell: First, 600 μL of a 2% (w/v) SLS solution was pipetted into the upper chamber and gently massaged into the skin using a cotton bud and the washings collected; this step was then repeated before further triplicate washings of the upper chamber with 600 μL of water. The total volume used in washings was 3 mL. The skin surface was dried with absorbent tissue, the Franz cell was then dismantled, and the skin was removed. For NIC, the skin was taken from the Franz cell directly after the patch was removed; as no adhesive residue was left on the skin, no further surface cleaning procedure was performed.

SC was sampled by tape-stripping. Strips of Scotch Book Tape 845 (3M, Bracknell, UK) were cut to approximately 2 × 2.5 cm, discharged of static electricity using an R50 discharging bar end ES50 power supply (Eltex Elektrostatik GmbH, Weil am Rhein, Germany) and weighed (Sartorius model SE2-F, Sartorius AG, Göttingen, Germany). Using a template, the exposed area of the SC was consistently collected by sequential application and removal of a maximum of 20 tapes. Tape strips were placed individually into 3 mL glass vials and subjected to overnight agitation in 1 mL of either a 1:1 v/v mixture of methanol and water for SA or a 3:2 v/v mixture of 10 mM pH 7.4 phosphate buffer and acetonitrile for NIC. To determine the amount of SA in the VT at the end of treatment, the exposed area of tape-stripped skin was trimmed, weighed (Sartorious BP 210 D, Göttingen, Germany), and agitated overnight in 5 mL of methanol. The final step was repeated twice using 3 mL of methanol to maximize SA recovery. The methanolic VT extracts were diluted 1:1 in water prior to analysis. For NIC, the tape-stripped skin, after trimming and weighing, was shaken overnight in 10 mL of the buffer/acetonitrile mixture. The final step was repeated (using 5 mL of mixture) to maximize NIC recovery.

#### Extraction Following Topical Application

Following the cleansing procedure already described, Ag/AgCl electrodes were positioned in Franz cell chambers without contacting the skin. For SA, the cathode and anode were located in the subdermal and upper chambers, respectively, and the latter was filled with 2 mL of 70 mM sodium chloride. For NIC, the position of the electrodes was reversed, and the upper chamber was filled with 1 mL of extraction solution comprising a 10 mM phosphate buffer containing 70 mM of either (i) NaCl buffered to pH 7.4 (achieved by a buffer composition of 8.09 mM Na_2_HPO_4_ and 1.91 mM NaH_2_PO_4_) or (ii) sodium gluconate buffered to pH 6.0 (achieved by a buffer composition of 1.21 mM Na_2_HPO_4_ and 8.79 mM NaH_2_PO_4_). It was hypothesized that (i) the latter would improve the efficiency of RI extraction because the gluconate ion, being less mobile than chloride, is a less competitive charge carrier to the NIC cations and (ii) that the lower pH would increase the percentage of ionised drug.

Following range-finding studies to determine a suitable duration of RI, a power supply (Yokogawa Programmable DC source, Tokyo, Japan) applied a direct current of 1 mA (0.5 mA cm^–2^) across the skin for 4 h (Table 1, experiment E2). Current application was interrupted at predetermined intervals, and 0.2 mL of receiver solution was sampled and replenished in SA experiments. In NIC experiments, the entire volume of receiver solution was sampled and replenished. A control experiment was performed which assessed the passive extraction (PE) of the drugs into 70 mM NaCl (unbuffered for SA and buffered to pH 7.4 for NIC; Table 1, experiment E3).

At the end of the extraction, the cells were disassembled, and the epidermal side of the skin was dried with absorbent tissue. Finally, the SC was sampled by tape-stripping, and the drugs were extracted from the tape and from the underlying VT as previously described.

#### Extraction from the Subdermal Chamber

The RI extraction of the two drugs from the subdermal compartment (SDC) into the relevant extraction solution (as described for E3, above) was characterized (Table 1, experiment E4) to assess the magnitude of the “subdermal contribution” compared to that measured after topical application of the formulations. The subdermal chamber was filled with a buffered solution containing a series of drug concentrations and, after a 1 h period of equilibration, a 1 mA current was imposed as explained above. The corresponding passive control experiment was performed using the highest subdermal concentration of SA assessed with RI and a NIC concentration of 0.9 M, *i.e.*, higher than any of those assessed with RI (Table 1, experiment E5).

### Experimental Procedure *In Vivo*

Six volunteers (for SA, four males and two females, ages between 19 and 27; for NIC, three males and three females, ages between 25 and 28) with no history of skin disease were enrolled into the study after providing informed consent. All procedures were approved by the Research Ethics Approval Committee for Health at the University of Bath (EP 22 043). The SA formulation was applied (5.2 μL cm^–2^) to three demarcated sites on the volar forearm of the volunteers’ choice for 24 h (as in the *in vitro* experiments). Each site was protected by a nonocclusive gauze. For NIC, three 2.27 cm^2^ sections cut from a Nicotinell patch were applied to the volar forearm for 4 h (the shorter application time compared to that used *in vitro* was more convenient for the participants and reduced their exposure to NIC). For both drugs, the volunteers were instructed to not wash their forearms or partake in any strenuous physical activity for the duration of the application period.

For both SA and NIC, tape-stripping was performed at an untreated skin site (i) to determine SC thickness using measurements of transepidermal water loss (TEWL) (AquaFlux AF200 probe, Biox Systems Ltd., London, UK) as previously reported;^43^ and (ii) to provide a negative control for drug analysis. To ensure adequate SC sampling and to comply with the approved protocol, tape-stripping was stopped when either 30 tapes had been used, TEWL had reached 60 g m^–2^ h^–1^, or TEWL was sixfold greater than the baseline (prestripping) measurement. In the SA study, one subject (male) out of six exhibited pronounced hypersensitivity to the tape-strip adhesive; as this precluded collection of a complete set of data, this volunteer was excluded from the study (reducing *n* from 6 to 5).

At the end of SA application, each treated site was wiped clean using two Sterets isopropyl alcohol 70% swabs. The SC was tape-stripped at one treated site, and the number of tapes used was guided by TEWL measurements described above. SA was extracted from tape strips using the same approach used for *in vitro* SC sampling, but, from the fourth tape onward, consecutive tapes were grouped and agitated in pairs to facilitate quantification. The same SC sampling procedure was adopted for NIC at the first treated site, except that TEWL measurements were not made because the occlusivity of the patch caused artificially elevated initial values. The number of tapes taken was, therefore, informed by that used at the untreated site.

**Figure 1:**
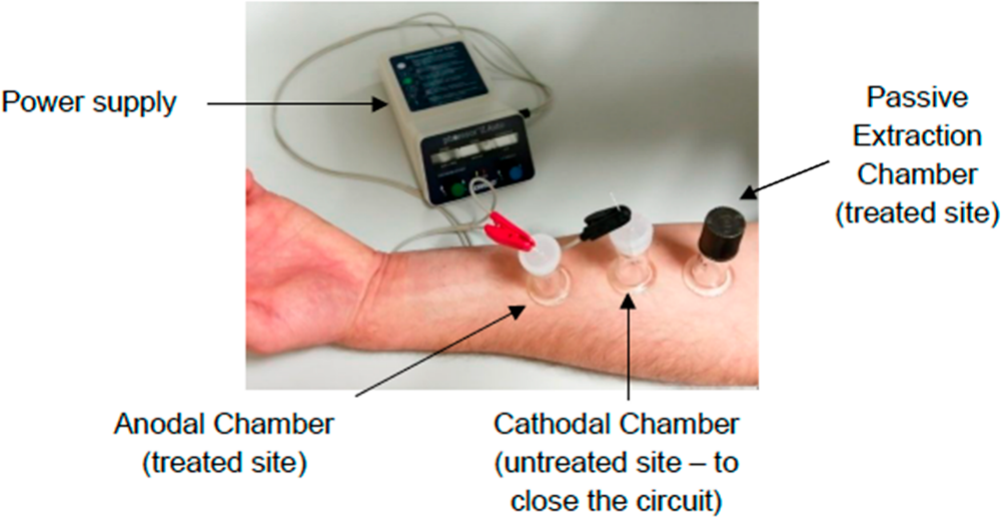
Set-up for the in vivo experiments showing (in this case) the disposition of the reverse iontophoresis chambers for SA, along with an adjacent chamber for passive extraction, on the ventral forearm.

The other skin sites treated with the SA or NIC formulations were subjected to a 4 h period of either RI or passive extraction. At each treated site and at one other adjacent position, glass chambers (contact area of 2.01 cm^2^) were secured onto the skin using Mefix medical adhesive (Mölnlycke, Gothenburg, Sweden) and a thin layer of silicone grease to ensure a good seal. For SA, the anode was placed in the chamber positioned at the site where the formulation had been applied (ensuring no contact with the skin); for NIC, the cathode occupied this chamber. The corresponding electrode of opposite charge was inserted into the second chamber at an untreated skin site near the first. The third chamber was positioned similarly to the first for passive extraction and contained no electrode (Figure 1).

For SA, the anode chamber was filled with 1.5 mL of 10 mM pH 6.0 phosphate buffer containing 40 mM NaCl; the chloride concentration was sufficient to sustain the electrochemistry involved in RI for the duration of current passage between sampling intervals. The same volume of the identical solution was dispensed into the passive extraction chamber. The cathode chamber was filled with 1.5 mL of 10 mM phosphate buffer (pH 6.0) containing 70 mM sodium gluconate. For NIC, the anode and cathode chambers contained exactly the same buffered solutions; the only difference from the setup for SA, of course, was that the cathode chamber was positioned at the site where the patch had been applied, while the anode was located in the chamber placed on an untreated skin site.

A portable FDA-approved medical device, the Iomed Phoresor II, was used to deliver a direct current of 1 mA for a period of 4 h; the current was interrupted at predetermined intervals to sample, and replenish with fresh buffer, a quantity of the “active” electrode contents (0.2 mL at the anode for SA and the entire cathodal volume for NIC) and the same volume of the passive extraction solutions; at the same time, the entire contents of the “nonactive” electrode chamber (cathode for SA, anode for NIC) were withdrawn and replenished with fresh solution.

### Analytical Chemistry

All SA and NIC samples were filtered through Minisart RC 0.45 μm syringe filters (Sartorius, Göttingen, Germany). An Ultimate 3000 ultrahigh pressure liquid chromatography system with UV detection (UHPLC-UV; ThermoFisher, Waltham, MA) was used for the quantification of both drugs. For SA, 20 μL volumes were injected on to a Supelco Ascentis Express column (5 cm × 2.1 mm, 2.7 μm particle size for *in vitro* or 10 cm × 2.1 mm, 2.7 μm particle size for *in vivo*) maintained at 50 °C. A gradient elution method was used: lines A and B were perfused at 0.6 mL min^–1^ with water and acetonitrile, respectively (each containing 0.1% v/v formic acid). To elute SA from the 5 cm column, line B was ramped from 20 to 100% between 0.1 and 1.1 min and maintained at 100% until 1.8 min. The column was re-equilibrated between 1.8 and 3.5 min. The 10 cm column was initially perfused with 2% line B at 0.6 mL min^–1^. The flow was reduced to 0.3 mL min^–1^ at 1 min and ramped back to 0.6 mL min^–1^ between 1 and 2 min. Between 2 and 4 min, the column was perfused with 20% line B, and between 5 and 8 min this was increased to 100%. Finally, the column was re-equilibrated with 2% line B until 10 min.

For NIC, volumes of 20 μL were injected on to a Kromatek HiQ Sil C18HS column (15 cm × 4.6 mm, 5 μm particle size) maintained at 25 °C. An isocratic method used a mobile phase consisting of a 30:35:35 v/v/v mixture of 10 mM pH 7.4 phosphate buffer, acetonitrile, and methanol, respectively, to elute NIC at 3.0 min. The flow rate was set to 1 mL min^–1^, and the analyte was detected at a wavelength of 260 nm.

### Data Analysis and Statistics

Unless otherwise indicated, values are reported as the arithmetic mean ± standard deviation. Extracted drug amounts are expressed as mass normalized per unit area of skin (nmol cm^–2^ or μmol cm^–2^) and extraction fluxes are defined in terms of mass per unit area per unit time (nmol cm^–2^ h^–1^ or μmol cm^–2^ h^–1^). Statistical analyses were performed using GraphPad Prism Version 9.0 (San Diego, CA) with the level of significance was set at α ≤ 0.05.

## Results

To compare the results from the *in vitro* experiments (Table 1, experiments E1–E3), the variations in SC depth sampled, VT thickness, and cumulative delivery to the subdermal fluid were assessed by one-way ANOVA, which found no significant interexperiment differences for either SA or NIC (Supplementary Tables S1–S3). Mass balances based on drug recovery from tape-strips, VT, subdermal fluid, and formulation (*i.e.*, unabsorbed active) accounted for 85–111% and 80–122% of the applied dose for SA and NIC, respectively (Supplementary Tables S1–S3).

### Skin Disposition after SA and NIC Application to Skin *In Vitro*

**Figure 2:**
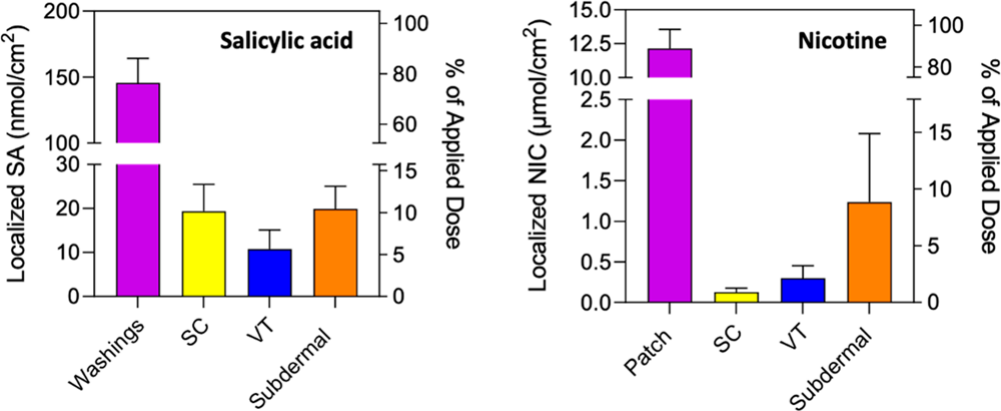
*In vitro* disposition of SA and NIC in skin after “passive loading” for 24 and 8 h, respectively (mean ± SD; *n* = 6 for SA and *n* = 8 for NIC).

*In vitro*, SA was delivered across the skin and into the SDC at an average flux of about 1 nmol cm^–2^ h^–1^ between 6 and 24 h. At the end of the application, the cumulative amount of SA in the subdermal compartment (SDC) was 20 ± 5.1 nmol cm^–2^ (Figure 2). SA localization in the SC after 24 h was approximately double that in the VT: 19 ± 6.1 versus 11 ± 4.3 nmol cm^–2^, respectively. As the mass, density (assumed to be 1 g mL^–1^),^55^ and exposed area of the VT are known, the concentration of SA in this skin compartment was calculated to be 80 ± 32 nmol mL^–1^. The complete 24 h data set is in Supplementary Table S1.

The corresponding average flux of NIC was 0.18 ± 0.10 μmol cm^–2^ h^–1^ (*i.e.*, 29 ± 17 μg cm^–2^ h^–1^) in the period between 2 and 8 h in exact agreement with the labeled Nicotinell input rate of a 10 cm^2^ patch delivering 7 mg/24 h. The cumulative delivery of NIC into the SDC in 8 h was 1.23 ± 0.84 μmol cm^–2^ (Figure 2), and the amounts recovered from the SC and VT were 0.13 ± 0.05 μmol cm^–2^ vs 0.30 ± 0.15, respectively; the latter corresponded to a calculated concentration of the drug in this compartment of 2.1 ± 1.0 μmol mL^–1^. The complete 8 h data set is summarized in Supplementary Table S1.

### RI and Passive Extraction of SA and NIC *In Vitro*

**Figure 3:**
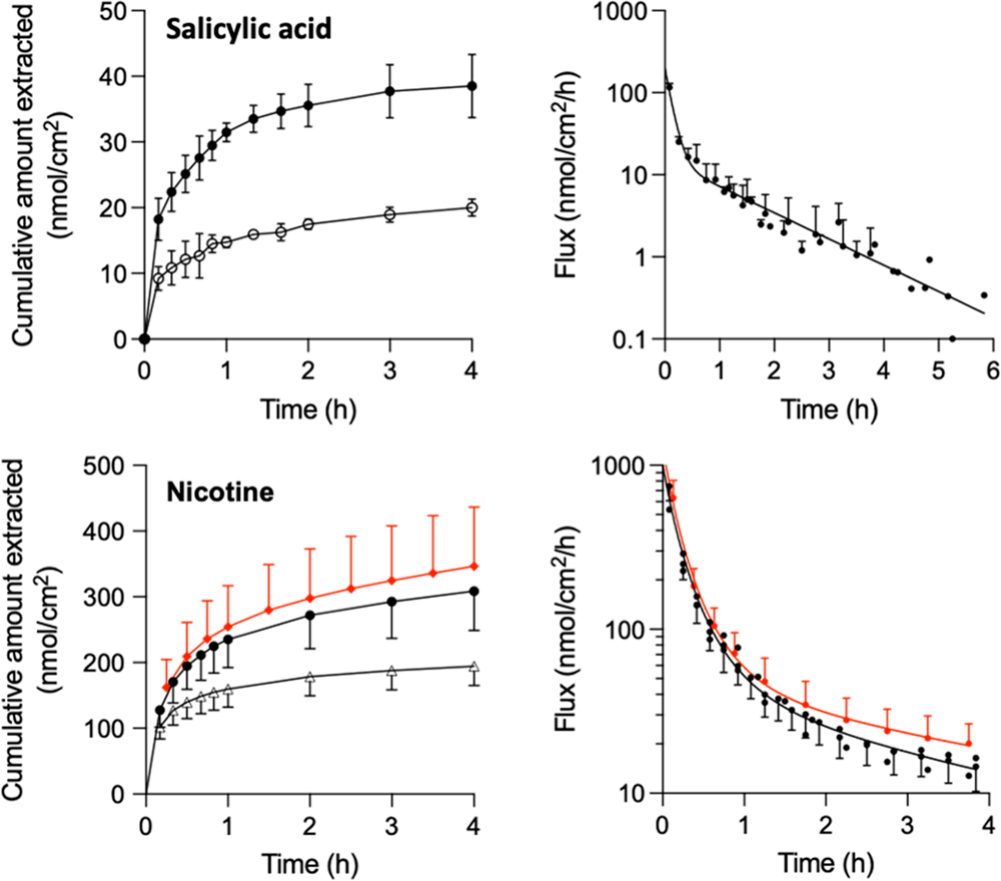
Noninvasive extraction from “loaded” skin *in vitro* with reverse iontophoresis (filled circles) and passively (open symbols) (left) and corresponding RI extraction fluxes (right). SA was extracted into a solution containing 70 mM NaCl (mean ± SD, *n* = 6); NIC was extracted into either a pH 7.4 buffer containing 70 mM NaCl (black symbols, *n* = 8) or (by RI only) into a pH 6.0 buffer containing 70 mM gluconate (red symbols, *n* = 6).

Immediately following applications of the SA and NIC formulations, drug extraction was performed either with or without current (experiments E2 and E3, respectively) over a 4 h period. The cumulative extraction profiles and the corresponding fluxes as a function of time are shown in Figure 3. For both drugs, statistically greater quantities were extracted using RI (*p* < 0.001; t test with Welch’s correction): 39 ± 5.7 nmol cm^–2^ of SA and, for NIC, 282 ± 40 and 346 ± 86 nmol/cm^–2^ into the chloride/pH 7.4 and gluconate/pH 6.0 buffers, respectively (the difference between the two NIC values was not statistically significant). Solvent extraction of SA and NIC from SC removed on the tapes and from the remaining tape-stripped skin at the end of RI confirmed that at least 80% of the drugs present in the skin after the “loading period (24 and 8 h, respectively, for SA and NIC) had been extracted by RI (Supplementary Table S2).

### Extraction of SA and NIC from the Subdermal Chamber *In Vitro*

**Figure 4:**
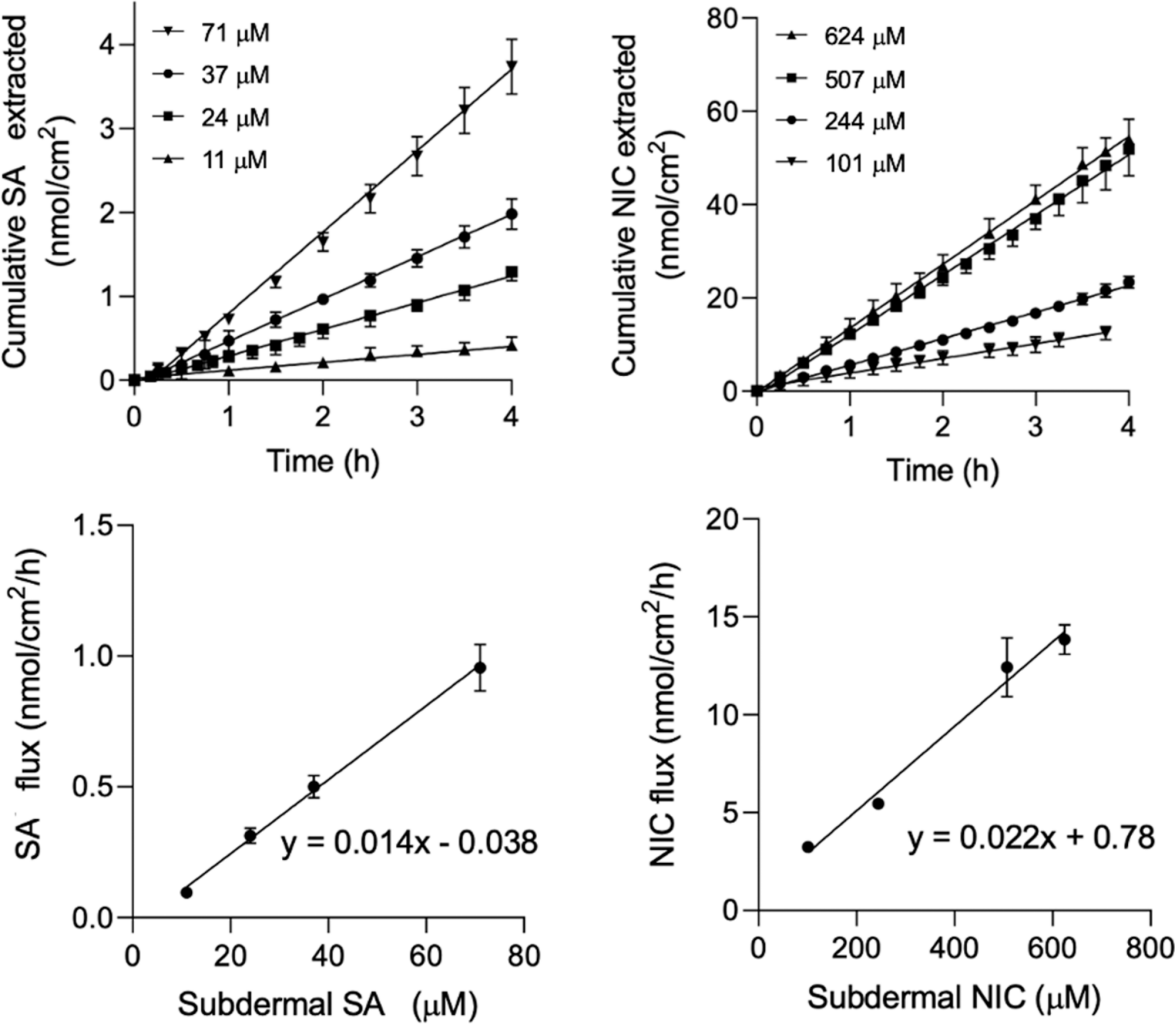
RI extraction profiles *in vitro* of SA and NIC (left and right upper panels, respectively) from the subdermal chamber as a function of time and the corresponding extraction fluxes as a function of concentration (lower panels) deduced from the linear portions of the extraction profiles (mean ± SD, *n* ≥ 3). The linear regressions shown had *R*^2^ > 0.95.

Both SA and NIC were extracted efficiently by RI (the former to the anode, the latter to the cathode), reaching steady-state within 1 h of current imposition over the ranges of subdermal concentrations investigated (experiment E4), as shown in Figure 4 (upper panels). The average extraction fluxes of the two drugs between 1 and 4 h was, as expected,^32,48^ directly proportional (*R*^2^ > 0.95) to the subdermal concentrations investigated, as described by the linear regressions in Figure 4 (lower panels).

From the RI extraction results, it was possible to calculate that SA carried between 0.0005% and 0.005% of the charge that was passed in 4 h; for NIC, the fraction was higher but never exceeded 0.1% (Supporting Information S4). Passive extraction of SA at the highest subdermal concentration used was undetectable after 4 h; for NIC, with a subdermal concentration of 0.90 mM, the average amount extracted without current was 2.0 ± 1.4 nmol cm^–2^; *i.e.*, ∼ 1/6th of that extracted by RI at the lowest subdermal concentration tested, confirming that the passive contribution to the RI data is negligible. It follows that, under the influence of electric current, the flux of both drugs is dominated by iontophoretic transport and that, in agreement with previous observations, the passive component can be neglected.^28,49^

### RI Extraction of SA and NIC from Human Volunteers *In Vivo*

**Figure 5:**
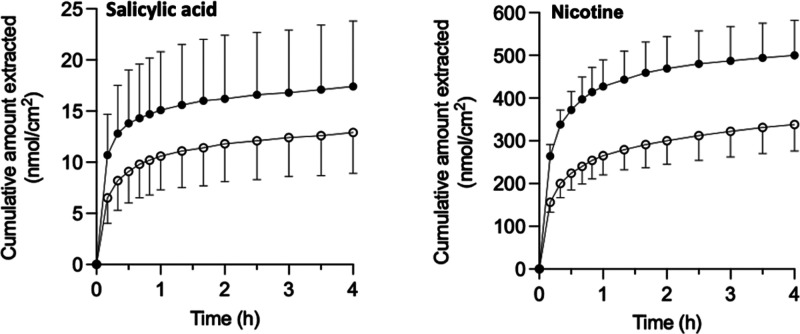
Noninvasive extraction of SA and NIC from “loaded” skin *in vivo* with reverse iontophoresis (filled circles) and passively (open symbols); Drugs were extracted into 10 mM pH 6.0 phosphate buffer containing (for SA) 40 mM NaCl or (for NIC) 70 mM sodium gluconate) (mean ± SD, *n* = 5–6).

After application of the SA and NIC formulations, extraction of the drugs was carried out with and without current for a 4 h period. The cumulative extraction profiles as a function of time are in Figure 5. As observed *in vitro*, statistically greater quantities of both SA and NIC were extracted using RI (*p* < 0.05; t test): 17 ± 6.4 nmol cm^–2^ of SA with current (versus 13 ± 4.0) nmol cm^–2^ without) and 500 ± 82 of NIC with RI (compared to 338 ± 62 nmol cm^–2^ passively).

### Mathematical Modeling of *In Vitro* and *In Vivo* RI data

**Figure 6:**
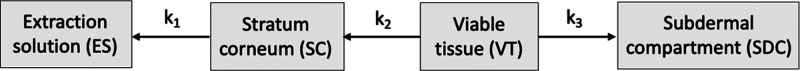
Schematic diagram of the compartmental model describing RI extraction immediately following a topical application period, where *k*_1_, *k*_2_, and *k*_3_ are first-order rate constants. *In vitro*, the subdermal compartment is the lower chamber of the Franz diffusion cell; *in vivo*, this compartment corresponds to the systemic circulation.

The experimental RI results for SA and NIC shown in Figures 3 and 5 suggest, at the least, a biphasic character that may be interpreted most simply as extraction of the two drugs first from the SC and then, subsequently, from beneath this barrier layer of the skin (*i.e.*, from the “viable” tissue (VT)). A simple compartmental model to describe this scenario is proposed in Figure 6.

At the start of the RI extraction, the initial, boundary conditions (*i.e.*, at *t* = 0) are:
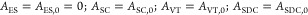
where *A*_ES_, *A*_SC_, *A*_VT_, and *A*_SDC_ are the amounts of drug in the extraction solution (ES), the stratum corneum (SC), the viable tissue (VT), and the subdermal compartment (SDC), respectively. The evolution of these quantities over the time of RI extraction is determined by solving the following simple differential equations for each compartment in the model:
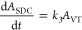
1
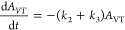
2
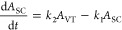
3
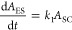
4This yields the results below:
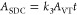
5
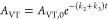
6
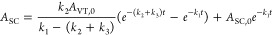
7
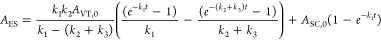
8The experimental *A*_ES_ profiles as a function of time of reverse iontophoresis, *in vitro* and *in vivo* for both SA and NIC, were analyzed using eq 8, and best-fit values of *A*_SC,0_, *A*_VT,0_, *k*_*1*_, and *k*_*2*_ were derived. For both drugs, *in vitro* and *in vivo*, the amount in the subdermal compartment was assumed to be zero for the duration of iontophoretic extraction (*i.e.*, *A*_SDC_ = *A*_SDC,0_ = 0). This is justified, *in vitro*, based on experimental observations for each drug. First, the linear relationship shown in Figure 4 for SA suggests that the maximum subdermal concentration achieved in the subdermal compartment at 24 h (∼7 μM) would result in a reverse iontophoretic flux of only about 0.05 nmol cm^–2^ h^–1^, that is, contributing less than 1% of the total amount extracted in 4 h. Similarly, for NIC, the data in Figure 4 suggest that, given the average subdermal concentration achieved after the 8 h application is ∼ 0.34 mM, the corresponding RI flux would not exceed 10 nmol cm^–2^ h^–1^ resulting in just about 10% of the quantity actually extracted in 4 h. Hence, the subdermal concentration for both drugs is sufficiently small (albeit nonzero) that the assumption *A*_SDC_ = *A*_SDC,0_ = 0 is reasonable.

*In vivo*, the assumption that the subdermal compartment is a perfect “sink” for both drugs is valid because of the substantial volumes of distribution of the two compounds in an adult. However, to generate the best-fit estimates for *A*_SC,0_, *A*_VT,0_, *k*_*1*_, and *k*_*2*_, both *in vitro* and *in vivo*, the value of *k*_*3*_ has to be “fixed”. *In vivo*, it is well-known that the transdermal delivery of nicotine enables an effective, target systemic concentration to be achieved and that *k*_*3*_ must therefore have a nonzero value. After a typical wear period of a nicotine patch, the plasma levels fall exponentially with a rate constant much smaller than that observed after intravenous administration of the drug; *i.e.*, “flip-flop” kinetics are operating,^50^ and the slower rate constant is describing the release, or desorption, of nicotine from the skin into the central pharmacokinetic compartment. In other words, this is equivalent to *k*_*3*_ in the model shown in Figure 6. A recent publication,^51^ which attempted to predict this drug clearance from the skin, reported an experimental measurement of *k*_*3*_ = 0.20 h^–1^, and this value of the rate constant was therefore fixed in the fitting of eq 8 to the NIC *in vivo* RI extraction data. For SA, a *k*_*3*_ value of 0.19 h^–1^ was selected on the basis of two independent studies in rats^57^ and in human^58^ volunteers^51^ following topical application of the drug. *In vitro*, the results for NIC clearly indicate that drug is transferred from the skin into the subdermal compartment during the period of RI (Supplementary Table S2) and the *in vivo* value of *k*_*3*_ (0.20 h^–1^) was again used. For SA, on the other hand, there was no measurable net transfer out of the viable tissue into the subdermal compartment during 4 h of RI (Supplementary Table S2), and best-fits to the profiles were therefore obtained with *k*_*3*_ set equal to 0 h^–1^.

**Figure 7:**
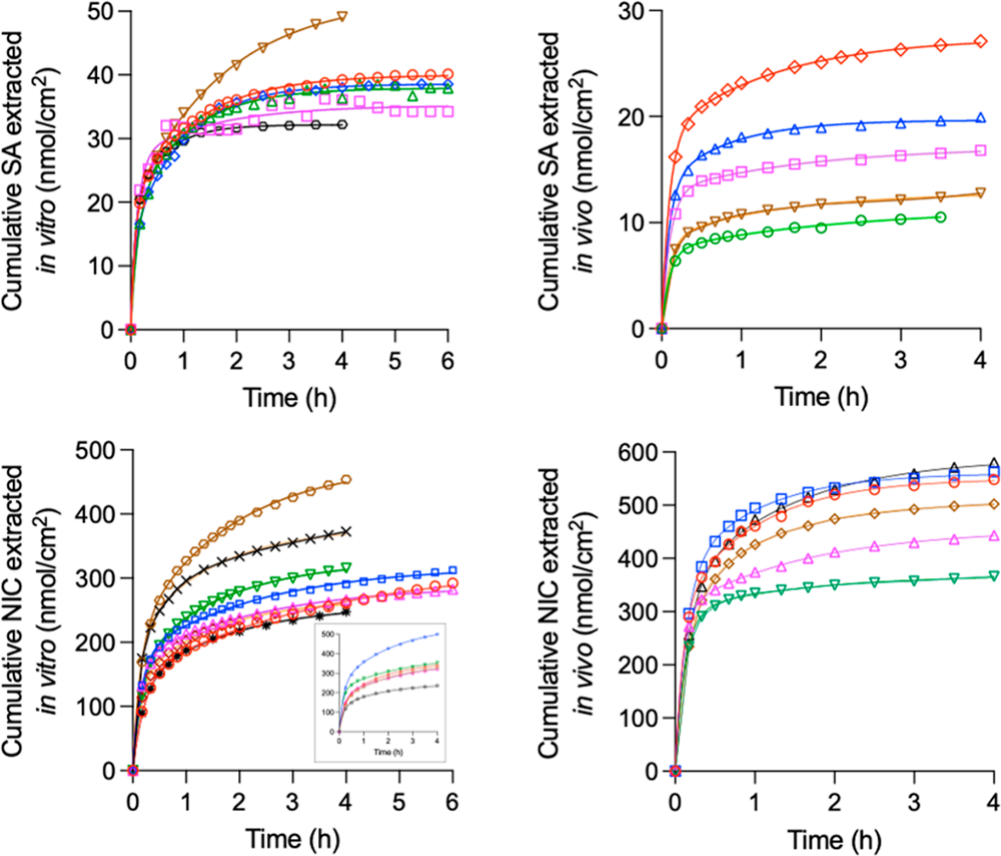
Individual RI extraction profiles of SA (upper panels) and NIC (lower panels) from “loaded” skin *in vitro* (left panels) and *in vivo* (right panels). SA was extracted into phosphate buffer containing NaCl, and NIC was extracted into phosphate buffer containing NaCl (*in vitro*) and sodium gluconate (*in vivo*) (mean ± SD, *n* = 5–8). The inset shows NIC extraction by RI *in vitro* into phosphate buffer containing sodium gluconate.

The compartmental analysis results are summarized in Table 2, and the best-fit estimates of the different parameters derived are compared to those measured experimentally. The individual data (and the best-fit profiles) for the replicate measurements *in vitro and in vivo* are in Figure 7, and complete data sets for each volunteer are in Supplementary Table S5.

**Table 2:** Analysis of the Experimentally Determined *A*_ES_ Profiles as a Function of Time of Reverse Iontophoresis *In Vitro* and *In Vivo* for Both SA and NIC using Equation 8 and the Derived Best-Fit Values of *A*_SC,0_, *A*_VT,0_, *k*_1_, and *k*_2_^a^

		*in vitro* (mean ± SD)		*in vivo* (mean ± SD)	
drug	parameter	experiment	model best-fit	experiment	model best-fit
salicylic acid^b^	*A*_SC,0_ (nmol cm^–2^)	19 ± 6.1	22 ± 3.6	16 ± 8.5^e^	13 ± 4.5
	*A*_VT,0_ (nmol cm^–2^)	11 ± 4.3	18 ± 8.5	n.d.^d^	7.3 ± 2.5
	*k*_1_ (h^–1^)	n.d.^d^	11 ± 2.6	n.d.^d^	10 ± 0.45
	*k*_2_ (h^–1^)	n.d.^d^	0.95 ± 0.39	n.d.^d^	0.54 ± 0.30
nicotine^b^,^c^,^f^	*A*_SC,0_ (nmol cm^–2^)	127 ± 49	188 ± 38^b^	453 ± 136^e^	335 ± 33
			199 ± 45^c^		
	*A*_VT,0_ (nmol cm^–2^)	300 ± 153	283 ± 156^b^	n.d.^d^	223 ± 75
			351 ± 118^c^		
	*k*_1_ (h^–1^)	n.d.^d^	6.4 ± 1.3^b^	n.d.^d^	8.5 ± 1.9
			5.8 ± 0.74^c^		
	*k*_2_ (h^–1^)	n.d.^d^	0.33 ± 0.15^b^	n.d.^d^	0.65 ± 0.18
			0.25 ± 0.09^c^		

a The value of *k*_3_ for NIC was set to 0.20 h^–1^*in vitro* and *in vivo*; for SA, the corresponding values were 0.19 and 0 h^–1^.

b RI extraction into chloride/pH 7.4 buffer (*n* = 6 for SA; *n* = 8 for NIC).

c RI extraction into gluconate/pH 6.0 buffer (*n* = 6 for NIC).

d Not determined.

e *n* = 5 for SA; *n* = 6 for NIC.

f Application times are 8 h *in vitro* and 4 h *in vivo*.

## Discussion

Proof-of-concept that reverse iontophoresis (RI) can noninvasively sample a topically applied drug from within the skin has been demonstrated *in vitro* and *in vivo*. The extracted amounts of SA and NIC as a function of time were then analyzed with a simple first-order compartmental model to provide estimates, and generate predictions, of drug quantities in the SC and in the deeper viable skin tissue (VT).

The initial *in vitro* experiments measured the disposition of SA and NIC in the SC, VT, and subdermal compartment (SDC) of the diffusion cell following 24 and 8 h applications, respectively, of the formulations used (Figure 2). Subsequently, after the same treatment protocols, the drugs were extracted into pH 7.4 phosphate-buffered saline at the skin surface (and the levels remaining in the SC, VT, and SDC were measured) following either 4 or 6 h periods of RI (or passive extraction) over the same period (Supplementary Tables S2 and S3). An attempt to improve the RI extraction of NIC by lowering the pH of the extraction medium to 6.0 and replacing chloride with the less mobile gluconate^52^ was unsuccessful (most likely because the change did not impact on the NIC cation’s ability to compete with the high concentration of Na^+^ present in the buffer).

The total RI extraction of SA was ∼40 nmol cm^–2^ (Figure 3), an amount that closely matched the combined recoveries in the SC and VT immediately following the 24 h application (Figure 2), demonstrating that the extraction had, in effect, removed essentially all of the drug taken up during the 24 h “loading” period (Supplementary Table S2). The total amount of NIC extracted by RI *in vitro* was ∼300–400 nmol cm^–2^ (Figure 3) and again confirmed that sampling was achieved from below the SC barrier. In contrast, the quantities of SA and NIC extracted passively were only about half of those extracted with current (Figure 3). The contributions of passive diffusion to the RI-accelerated extraction of the two drugs is primarily due to an initial burst “release” from the outer SC, a phenomenon previously reported for a number of endogenous compounds, including amino acids,^36^ lactate,^28^ glucose,^34,35,53^ and urea.^35,54^

Subsequent *in vivo* studies in human volunteers then showed the feasibility of applying RI to sample drugs in a practical, real-world scenario (Figure 5). Variability in the results for NIC was comparable to that observed *in vitro* (% CVs were 21% and 16%, respectively); for SA, the *in vivo* variability (% CV = 31%) was greater than that seen *in vitro* (% CV = 15%). Overall, these results are consistent with those in the literature, despite the fact that the *in vitro* results were acquired using skin from only a single animal.

Deconvolution of the RI extraction profiles to resolve the individual quantities of drug extracted from the SC and from the VT was achieved by applying a simple linear kinetic model (Figure 6) to describe the biphasic nature of the data (as indicated in Figure 3). A key assumption of the approach was to assume that the amounts of the two drugs in either the subdermal chamber of the *in vitro* diffusion cell, or in the body *in vivo*, did not change significantly during RI and that any extraction from these compartments was therefore negligible. The justification for this approximation is based on the fact that the salicylate anions and the NIC cations carry relatively small fractions of the iontophoretic current applied (the major responsibility falling to more mobile species available, *i.e.*, Cl^–^ and Na^+^, respectively) and meaning that the electrotransport of SA and NIC would be directly proportional to their subdermal concentrations.^33,48^ The experimental observations reported in Figure 4 (and Supplementary Tables S2 and S4) fully support this contention.

The ability to noninvasively predict the skin disposition of SA and NIC was first tested by comparing the experimentally determined quantities of the drugs after the “loading” periods in the SC (assessed by tape-stripping) and the VT (measured by extraction of the actives from the tape-stripped skin) with best-fit model values to the RI data (Table 2). *In vitro*, the deduced amounts of both drugs in the SC and in the VT were in excellent agreement (*i.e.*, all within less than a factor of 2) with the experimental values. As expected, given the extraction profiles, the first-order rate constant (*k*_*1*_) describing drug transfer from the SC to the extraction solution under RI was about an order of magnitude greater than that (*k*_*2*_) representing drug movement from the VT to the SC (Table 2).

From the *in vitro* experiments, the average concentration of drug in the VT at the beginning of the extraction period (*C*_VT,0_) can be calculated from the measured and best-fit area-normalized amounts (*A*_VT,0_) of SA and NIC using eq 9 (see Table 3):
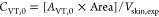
9where *Area* = 2.01 cm^2^ and *V*_*exp*_ is determined for each sample as the ratio of the mass of the tape-stripped skin to its density of 1 g mL^–1^.^55^

**Table 3:** Conversion of the RI-Measured or Deduced Quantities of SA and NIC in the Viable Tissue (VT) of the Skin below the SC into Estimated Concentrations^a^

	*in vitro C*_VT,0_ (μmol mL^–1^)		*in vivo C*_VT,0_ (μmol L^–1^)^e^	
drug	experiment	model best-fit	experiment	model best-fit
salicylic acid	0.080 ± 0.032	0.12 ± 0.059	n.d.^d^	0.084 ± 0.029
nicotine	2.14 ± 1.03	1.80 ± 1.09^b^	n.d.^d^	1.07 ± 0.36
		2.15 ± 0.92^c^		

a See the text for details.

b RI extraction into chloride/pH 7.4 buffer (*n* = 6 for SA; *n* = 8 for NIC).

c RI extraction into gluconate/pH 6.0 buffer (*n* = 6 for NIC).

d Not determined.

e *n* = 5 for SA; *n* = 6 for NIC.

To transform the *in vivo* best-fit values of *A*_VT,0_ to an average concentration, the steady-state volume of distribution (*V*_exp_) of the exposed area of skin was estimated using a recently reported method (eq 10):^51^
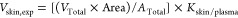
10where *V*_Total_ and *A*_Total_ have values of 2.6 L and 1.8 m^2^, respectively, for an individual weighing 70 kg. The skin/plasma partition coefficient (*K*_skin/plasma_) is computed using eq 11 as follows:^51,56^
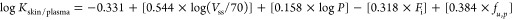
11where *V*_ss_ is the systemic volume of distribution at steady state, *F*_i_ is the ionised fraction of drug at pH 7.4, and *f*_*u,p*_ is the fraction of drug bound to plasma proteins.

Using literature values for SA (*V*_ss_ = 8.6 L,^46^ log *P* = 2.3,^40^*F*_i_ = 1.0, and *f*_*u,p*_ = 0.15),^47^ the estimated value of *K*_skin/plasma_ is 0.63; substitution into eq 10 then generates the result that *V*_skin,exp_ = 0.17 mL. For NIC, the calculation of *V*_skin,exp_ (undertaken in the same way) had already been reported to be 0.42 mL.^51^ With these values, the experimental and model best-fit values of *A*_VT,0_ reported in Table 2 for the two drugs can be converted into the corresponding concentrations (*C*_VT,0_) using eq 9.

The results are presented in Table 3 (note that, *in vivo*, self-evidently, no measurement of *A*_VT,0_ was performed, but conversion of the model best-fit values deduced from the RI data can provide estimates of *C*_VT,0_). The agreements between experiment and model *in vitro*, and between the model best-fits *in vitro* and *in vivo*, are considered to be more than reasonable given the preliminary nature of the study undertaken. This is reinforced by the fact that the *in vitro* experiments employed porcine skin, while the *in vivo* study was conducted in healthy human volunteers.

## Conclusions

The results of this research demonstrate the feasibility of RI to efficiently extract two drugs from different skin compartments following delivery from either a topical or transdermal formulation. Proof-of-concept is based on data from salicylic acid and nicotine, active compounds that, under physiological conditions, are net negatively and positively charged, respectively. Initial *in vitro* experiments provided cumulative extraction vs time profiles which, when analyzed with a relatively simple compartmental pharmacokinetic model, permitted the quantities that had been delivered into the SC and VT to be deduced and the drug concentrations in the “living” skin to be estimated; agreement with experimental measurements, in terms of quantity and concentration, was excellent. The derived first-order rate constants describing transfer between the VT and SC, and between the SC and the extraction solution, were consistent with the relatively rapid depletion of drug from the SC followed by a slower transfer out of the VT. Successful translation of the RI approach *in vivo* was achieved, which enabled accurate predictions of SC-localized drugs (confirmed by direct analysis using tape-strip sampling) and showed that the estimated VT amounts and concentrations *in vivo* were acceptably similar to the *in vitro* values.

Clearly, more work is needed to better understand the scope and limitations of the method. For instance, the implementation of the technique to provide more details about drug concentration versus depth and time profiling and the more general applicability to a wide range of dermatological drugs, the physicochemical properties of which may be less compatible with the use of iontophoresis (such as lipophilic corticosteroids and retinoids of limited water solubility), requires critical examination. That having been said, a clear benefit of RI as a tool with which to assess topically applied drug bioavailability in the skin is the relatively noninvasive manner (as opposed to a biopsy, for example) by which both SC and VT localization can be quantified *in vivo*. The approach would also lend itself to the evaluation of cutaneous drug disposition once steady-state levels in the skin compartment have been achieved via repeated dosing; this could be important when comparing formulations that differ in their “inactive” ingredients, the impact of which on skin barrier function may require time to become evident.

## Supporting Information Available

Summary of compartmental recoveries after 24 h (SA) or 8 h (NIC) of topical application (E1), summary of compartmental recoveries after RI extraction following 24 (SA) or 8 h (NIC) of topical application (E2), summary of compartmental recoveries after PE following 24 (SA) or 8 h (NIC) of topical application (E3), summary of iontophoretic fluxes and transport numbers derived using data generated in the SDC extraction experiment series (E4), and summary of *in vivo* experimental data (PDF)
