# Correction to “Reverse Iontophoresis: Noninvasive Assessment of Topical Drug Bioavailability”

**DOI:** 10.1021/acs.molpharmaceut.4c00176

**Published:** 2024-03-05

**Authors:** Kieran Moore, Sébastien Grégoire, Joan Eilstein, M. Begoña Delgado-Charro, Richard H. Guy

The units of *In vitro C*_*VT,0*_ and *In vivo C*_*VT,0*_ in Table 3 should both be μmol mL^–1^. The results, discussions, and conclusions in the original article are not affected by this correction.

