# Supplementary material for: Reverse Iontophoresis: Noninvasive Assessment of Topical Drug Bioavailability
Source: Mol Pharm. 2023 Dec 7;21(1):234–44. doi: 10.1021/acs.molpharmaceut.3c00791 (PMC10762657; doi:10.1021/acs.molpharmaceut.3c00791)
Supplement: Supplementary file 1 — mp3c00791_si_001.pdf [file mp3c00791_si_001.pdf]

Reverse iontophoresis: non-invasive assessment of topical drug bioavailability

Kieran Moore[1], Sébastien Grégoire[2], Joan Eilstein[2],

M. Begoña Delgado-Charro[1] and Richard H. Guy[1,*]

[1]University of Bath, Department of Life Sciences, Claverton Down, Bath, BA2 7AY, U.K.

[2]L'Oréal Research and Innovation, 1 Av. Eugène Schueller, 93600 Aulnay-sous-Bois, France

*Correspondence: r.h.guy@bath.ac.uk

Supplementary Table S1 - Summary of compartmental recoveries after 24-hour (SA) or 8-hour (NIC) topical/transdermal application (E1).

Replicate nº	SC (nmol/cm^2)	VT (nmol/cm^2)	SDC[$] (nmol/$cm^2$)	Washings (SAL; nmol/cm^2) or Patch (NIC; µmol/ cm^2)	Recovery (%)	SC depth sampled (µm)	Mass of tape-stripped skin (mg)	VT concentration (nmol/mL)
				Salicylic Acid				
1	15.3	4.9	15.6	152	99.0	13.9	176.0	56.1
2	14.4	12.1	18.0	162	108.7	18.0	218.4	111.0
3	12.8	10.3	18.6	169	110.7	8.81	293.8	70.4
4	21.7	9.3	23.5	141	93.8	12.0	310.3	60.4
5	23.8	18.1	15.4	121	85.1	15.3	279.6	130.0
6	28.2	10.1	28.6	130	106.7	20.5	381.7	53.3
Mean	19.4	10.8	19.9	146	100.7	14.8	276.6	80.2
SD	5.1	4.3	5.1	18.5	10.0	4.2	72.1	32.3
				Nicotine				
1	75.5	149	1290	11.5	92.9	12.9		
2	83.7	301	2802	10.3	96.3	17.9	n.d.*	
3	86.8	306	2109	11.5	100.6	15.0		
4	166	611	1186	14.8	119.9	13.5	320	3838
5	128	181	362	12.5	94.4	8.1	320	1137
6	96.0	234	380	13.2	99.9	9.5	287	1634
7	183	423	762	11.2	90.2	20.6	385	2208
8	199	198	1005	12.3	98.3	21.1	214	1858
Mean	127	300	1237	12.2	99.1	14.8	305	2135
SD	49.1	153	844	1.4	9.1	4.8	62.1	1028

[$]SDC = subdermal compartment. [*]n.d. = not determined

Supplementary Table S2 – Summary of compartmental recoveries after RI extraction following 24-hour topical (SA) or 8-hour transdermal (NIC) application (E2).

Replicate nº	Extraction duration (h)	SC (nmol/cm^2)	VT (nmol/cm^2)	SDC 24 h (nmol/cm^2)	SDC post-extraction (nmol/cm^2)	Washings (SAL nmol/cm^2) or Patch (NIC; µmol/ cm^2)	ES* (nmol/cm^2)	Recovery (%)	SC depth sampled (µm)	Mass of tape-stripped skin (mg)	VT concentration (nmol/mL)
Salicylic Acid											
1	6	0.9	1.2	7.8	6.8	132	40.1	90.3	24.0	216	11.4
2	6	0.8	1.3	16.4	17.8	131	38.0	92.7	17.8	309	8.2
3	6	1.3	1.8	8.4	8.8	135	34.3	89.4	20.2	327	11.3
4	6	1.3	0.8	18.6	20.2	125	38.6	99.9	12.2	335	4.7
5	4	7.2	2.3	15.4	15.7	158	32.2	103.4	10.1	339	13.6
6	4	9.8	3.3	13.4	13.2	115	49.1	91.0	15.0	316	21.1
Mean		3.6	1.8	13.3	13.8	133	38.7	94.5	16.6	307	11.7
SD		3.9	0.9	4.4	5.2	14.4	5.9	5.8	5.2	46	5.5
Nicotine (Chloride/pH 7.4 Extraction Solution)											
1	6	7.8	41.2	1144	1214	13.8	292	110.0	14.6	288	287
2	6	5.3	35.7	Contamination	Contamination	11.2	312	86.4	14.3	343	209
3	6	4.8	35.7	873	911	12.1	283	95.3	16.5	252	285
4	4	9.8	79.4	590	733	15.8	316	121.3	9.3	340	470
5	4	5.4	54.1	493	541	13.0	262	99.1	7.9	317	343
6	4	4.5	153	1565	1684	11.5	454	98.7	11.7	428	719
7	4	11.5	63.2	609	787	12.0	248	93.7	13.8	345	368
8	4	5.7	53.3	1042	1198	11.5	373	94.4	13.9	269	399
Mean		6.9	64.5	876	1010	12.6	318	99.9	12.8	323	385
SD		2.6	38.7	367	385	1.6	67.1	10.9	2.9	55.3	156
Nicotine (Gluconate/pH 6.0 Extraction Solution)											
1	4	5.7	72.5	1380	1385	10.8	326	89.9	19.1	387	377
2	4	4.9	98.5	1279	1491	10.3	500	88.8	9.5	382	519
3	4	10.6	52.3	1131	1201	11.3	321	92.3	16.6	308	342
4	4	5.0	39.5	1111	1163	10.7	353	87.6	14.5	224	354
5	4	1.7	62.8	831	953	10.6	340	85.4	9.5	387	327
6	4	1.4	44.2	671	718	10.2	236	80.1	12.3	392	227
Mean		4.9	61.6	1067	1153	10.6	346	87.4	13.6	347	357
SD		3.4	21.7	269	283	0.4	86.0	4.2	3.9	68.0	95

*ES = extraction solution

Supplementary Table S3 – Summary of compartmental recoveries after passive extraction following 24-hour (SA) or 8-hour (NIC) topical/transdermal application (E3).

Replicate nº	Extraction duration (h)	SC (nmol/cm^2)	VT (nmol/cm^2)	SDC 24 h (nmol/cm^2)	SDC post-extraction (nmol/cm^2)	Washings (SAL nmol/cm^2) or Patch (NIC; µmol/cm^2)	ES (nmol/cm^2)	Recovery (%)	SC depth sampled (µm)	Mass of tape-stripped skin (mg)	VT concentration (nmol/mL)
Salicylic Acid											
1	6	1.6	3.6	28.7	30.7	118	19.2	87.5	22.4	348	20.7
2	6	4.2	2.5	13.3	17.6	130	20.2	88.4	26.8	258	19.1
3	6	1.8	2.4	10.5	13.6	153	21.9	97.6	18.5	239	19.7
4	4	10.3	3.2	25.6	30.1	142	21.6	102	20.4	310	20.6
5	4	11.2	3.7	23.9	26.5	118	20.8	88.0	14.9	135	55.4
6	4	9.3	9.4	25.7	29.9	97.0	19.7	91.6	18.7	395	47.8
Mean		6.4	4.1	21.3	24.7	126	20.6	92.4	20.3	281	30.6
SD		4.4	2.6	7.5	7.3	19.9	1.1	5.8	4.0	92	16.5
Nicotine (Chloride/pH 7.4 Extraction Solution)											
1	6	5.9	28.2	346	380	11.3	190	85.6	9.5	236	240
2	6	4.5	60.2	594	875	11.0	160	87.0	6.7	329	368
3	6	8.1	56.9	610	861	11.8	155	92.2	9.2	397	288
4	4	11.7	90.8	753	924	15.8	221	122	9.5	323	565
5	4	11.9	70.7	876	1144	12.0	220	96.7	7.4	352	403
6	4	4.3	81.2	1290	1680	11.8	226	98.8	11.7	384	425
7	4	11.7	85.7	1021	1407	11.9	232	97.8	22.6	327	527
8	4	15.8	52.1	604	837	12.8	174	99.4	13.7	306	343
Mean		9.2	65.7	762	1014	12.3	197	97.5	11.3	332	395
SD		4.2	20.7	295	396	1.5	31.4	11.3	5.1	49.8	111

Supplementary Table S4 – Summary of iontophoretic fluxes and transport numbers derived from data generated in the SDC extraction experiment series (E4; mean ± SD; *n* = 3).

Drug	Subdermal Concentration (μM)	Subdermal Mole Fraction ($\times 10^4$)	Flux ($nmol/cm^2/h$)	Transport Number ($\times 10^6$)
Salicylic acid	11	0.75	0.09 (± 0.02)	5.05 (± 1.10)
	24	1.60	0.31 (± 0.01)	16.5 (± 0.49)
	36	2.39	0.50 (± 0.04)	27.2 (± 2.39)
	71	4.66	0.97 (± 0.09)	52.4 (± 5.02)
Nicotine	101	6.26	3.15 (± 0.21)	169 (± 11.3)
	244	15.1	5.67 (± 0.34)	305 (± 18.3)
	507	31.3	12.87 (± 1.30)	693 (± 69.8)
	624	38.4	13.69 (± 0.72)	738 (± 39.0)

Supplementary Table S5 – Summary of *in vivo* experimental data

Drug	Subject	Baseline TEWL ($g.m^{-2}.h^{-1}$)	Control tape-stripped site: Nº of Tapes Used	Control tape-stripped site: SC Depth Sampled (µm)	Control tape-stripped site: Final TEWL ($g.m^{-2}.h^{-1}$)	Control tape-stripped site: Modelled SC Thickness (µm)	Test tape-stripped site: Nº of Tapes Used	Test tape-stripped site: SC Depth Sampled (µm)	Test tape-stripped site: Final TEWL ($g.m^{-2}.h^{-1}$)	Test tape-stripped site: SAL in SC ($nmol/cm^2$)	Non-invasive extraction ($nmol/cm^2$): SAL in ES (RI)	Non-invasive extraction ($nmol/cm^2$): SAL in ES (PE)
Salicylic acid	A	7.2	18	11.1	55.4	11.9	30	11.4	80.7	29.1	27.1	18.9
	B	10.6	19	6.56	62.9	7.2	21	[a] n.d.	60.1	16.1	[b] 10.5	8.9
	C	15.4	21	5.4	66.4	6.9	26	6.8	60.8	16.2	19.9	14.2
	D	16.5	27	6.4	63.6	6.7	28	7.9	72.7	14.7	16.8	12.9
	E	13.4	24	3.3	63.8	3.7	24	3.6	67.7	5.3	12.8	9.5
	Mean	12.6	21.8	6.6	62.4	7.3	25.8	7.4	68.4	16.3	19.2	12.9
	SD	3.8	3.7	2.9	4.1	2.9	3.5	3.2	8.6	8.5	6.0	4.0
Nicotine	A	7.3	17	6.8	50.7	7.2	29	12.6		519	548	299
	B	7.1	28	7.8	44.0	8.9	14	9.5		665	563	421
	C	15.4	21	5.4	60.8	5.9	15	5.3	[c] n.d.	279	366	308
	D	12.8	29	4.6	62.4	4.8	20	8.4		362	443	277
	E	8.7	23	7.3	56.7	7.6	23	12.1		400	502	[d] 286
	F	9.1	28	8.4	54.1	13.3	28	10.9		490	580	385
	Mean	10.1	24.3	6.7	54.8	8.0	21.5	9.8		453	500	338
	SD	3.3	4.8	1.5	6.8	3.0	6.3	2.7		136	82	62

[a] Not determined due to a problem with calibration of the balance.
[b] Sampling terminated at 3.5 h due to a leak from one chamber on the skin.
[c] TEWL not determined post-patch application.
[d] Sampling terminated after 1h 20 min due to a leak in this chamber.
